# With or Without a System: How Category‐Specific and System‐Wide Cognitive Biases Shape Word Order

**DOI:** 10.1111/cogs.70139

**Published:** 2025-11-26

**Authors:** Annie Holtz, Simon Kirby, Jennifer Culbertson

**Affiliations:** ^1^ Department of Linguistics and English Language The University of Edinburgh

**Keywords:** Cognitive biases, Typology, Silent gesture, Regularization, Word order

## Abstract

Certain recurrent features of language characterize the way a whole language system is structured. By contrast, others target specific categories of items within those wider systems. For example, languages tend to exhibit consistent order of heads and dependents across different phrases—a system‐wide regularity known as harmony. While this tendency is generally robust, some specific syntactic categories appear to deviate from the trend. We examine one such case, the order of adjectives and genitives, which do *not* exhibit a typological tendency for consistent order with respect to the noun. Instead, adjectives tend to follow and genitives precede the noun. Across two silent gesture experiments, we test the hypothesis that these category‐specific ordering tendencies reflect cognitive biases that favor (i) conveying objects before properties that modify them, but (ii) conveying expressions of possession before possessors. While our hypothesis is thus that these biases are semantic in nature—they impact preferences for how concepts are ordered—the claim is that they may have downstream effects on conventionalized syntax by contributing to an over‐representation of postnominal adjectives and prenominal genitives. We find that these biases affect gesture order in contexts where no conventionalized system is in place. When a system *is* in place, participants learn that system, and category‐specific biases do not impact their learning. Our results suggest that different contexts reveal distinct types of cognitive biases; some are active during learning and others are active during language creation.

## 1 Introduction

Typological research has revealed a variety of regularities in how languages order meaningful elements, such as affixes, words, and phrases (Coons, 2022; Dryer, 1992; Greenberg, 1963; Hawkins, 1990). The underlying causes of these typological patterns remain an open question in linguistics, but there are several proposed explanations. These include innate constraints on language structure (Chomsky, 1993; Travis, 1984), lineage‐specific trends in language history (Dunn, Greenhill, Levinson, & Gray, 2011; Piantadosi & Gibson, 2014), common processes of language change (Bybee, 2008; Collins, 2019), and cognitive biases which tend to (dis)favor certain linguistic structures, such as the order of linguistic elements in a phrase or specific phonological rules (Culbertson, Smolensky, & Legendre, 2012; Finley, 2015, 2018; Martin, Holtz, Abels, Adger, & Culbertson, 2020; Saldana, Oseki, & Culbertson, 2021; Wilson, 2006). Research examining the latter type of explanation has explored the role that cognitive biases play in a variety of linguistic phenomena, including phonology (Finley, 2015; Martin & White, 2019; Wilson, 2006), morphology (Saldana et al., 2021), basic word order (Goldin‐Meadow, So, Özyürek, & Mylander, 2008; Hall, Mayberry, & Ferreira, 2013), and syntactic harmony (i.e., consistent order between heads and dependents, Christiansen, 2000; Culbertson, Franck, Braquet, Barrera Navarro, & Arnon, 2020; Culbertson et al., 2012).

These studies use controlled experiments to test whether linguistic patterns that are more common across languages are preferred by participants. In some cases, the preferences targeted are at the level of an individual word, phrase, or utterance. For example, research on basic word order typology has proposed a subject‐first bias (Futrell et al., 2015; Meir et al., 2017). This bias targets a specific category with in an utterance, thus for any given utterance that includes this category, one can ask whether the bias is satisfied (i.e., that subject is placed first) or not.^1^ In other cases, the preferences targeted are features of a language *system* that hold across phrases or utterances. For example Culbertson et al. (2020) show that learners prefer placing modifiers in a consistent position across phrases in a language. This bias targets a more abstract category type (i.e., dependents), and crucially, whether or not it holds can not necessarily be assessed based on a single phrase. While a single phrase *might* contain multiple instances of heads and/or dependents, a complete picture of harmony comes by looking across different phrases, that is, describes a more general feature of a language system, like dependents come first, or dependents come last. Here, we will refer to the former type of preference as *category‐specific*, and the second as *system‐wide*. System‐wide and category‐specific biases have been found in multiple linguistic domains. In some cases though, they can come into conflict. Here, we use such a case of potential conflict to explore how, and under what circumstances category‐specific and system‐wide biases come to influence language typology. But first, we will define and provide additional examples of these two types of biases.

### 1.1 Category‐specific and system‐wide biases

Category‐specific biases target particular categories of linguistic items (e.g., sounds, words) within a given unit (e.g., word, phrase, sentence). In principle, the level of abstraction of the category may differ depending on the bias; it might target specific sounds, or words (e.g., the sound “t,” or the adjective “big”), or abstract categories like coronals, adjectives; or even consonants. What distinguishes this type of bias is that it applies at the level of that category in a given context, without the need to reference to any other aspect of the system. For example, as mentioned above, the “subject‐first” bias describes the preference to place syntactic subjects first in a sentence. Thus, this type of bias targets a particular category—subjects—in a particular context, a sentence. In any given sentence, we can ask whether the bias is satisfied, or not, by examining if the initial element in the sentence is the subject. However, importantly, for us, this bias has been argued to derive from the association between the syntactic role of subject and a semantic feature, for example, agency or animacy. Thus, the label “subject‐first” is potentially misleading, and a more accurate label might be, for example, “agent‐first,” reflecting the claim that the relevant bias applies to a specific *semantic* category rather than a syntactic one (Futrell et al., 2015; Goldin‐Meadow et al., 2008; Meir et al., 2017). Independently of whether this bias is formulated in terms of a semantic or syntactic category, whether a given utterance conforms to the bias can be assessed without reference to any other utterances or any other aspects of the system. Put another way, the claim is that the influence of this kind of bias on language users' behavior happens on an utterance‐by‐utterance basis. In contrast, system‐wide regularity—that is, whether a language system is consistent in having subjects or agents first, or last—is, we claim, governed by a distinct bias operating only once sufficient evidence of the system is present, more akin to harmony (following Culbertson & Kirby, 2016; Motamedi, Wolters, Naegeli, Kirby, & Schouwstra, 2021).

In addition to specific semantic characteristics of an entity, (e.g., agency, animacy, salience) (Gibson et al., 2013; Hall et al., 2013; Kirton, Kirby, Smith, Culbertson, & Schouwstra, 2021; Meir et al., 2017), other studies have argued that semantic features of individual actions or events influence ordering preferences. Schouwstra and de Swart (2014) found that both Dutch‐ and Turkish‐speaking participants preferred to condition gesture order on the type of event: extensional meanings like *throw* or *carry* led to gestures orders with the agent first and verb last (more similar to SOV), but intensional events like *hear* or *dream of* led to gesture orders with the agent first and the verb medial (more like SVO) (see also Motamedi et al., 2021). This difference in preferred order for conveying extensional and intensional events targets specific semantic categories, and can be assessed on the basis of an individual instance of that category in its context; thus, it is another example of a category‐specific bias.

A somewhat more complex example of a category‐specific bias has been argued to influence noun phrase word order. There is a typological tendency for adjectives to come closer to the noun than numerals, and for numerals to come closer to the noun than demonstratives (Dryer, 2018). Culbertson and Adger (2014) provide experimental evidence for a bias which aligns with this tendency. In this case, there are two relevant categories (two types of nominal dependents). Nevertheless, to determine whether a given noun phrase conforms to this bias or not, it suffices (indeed, it is necessary) to consider an individual instance of a noun phrase. For example, an instance of Noun‐Adj‐Num‐Dem adheres to the bias, whereas an instance of Noun‐Num‐Dem‐Adj does not. These orders are argued to be preferred because they transparently reflect, or are *homomorphic* to a semantically motivated single representation in which adjectives (descriptive properties) are grouped most closely with nouns (entities), and demonstratives (locations) furthest away (see, e.g., Culbertson & Adger, 2014; Dryer, 2018; Martin et al., 2020; Rijkhoff, 2004). Nevertheless, according to our definition, this is a category‐specific bias, since it targets specific categories within a given unit of language.^2^

The bias in favor of homomorphic word order in the noun phrase bias has been tested both using artificial language learning (ALL) and silent gesture paradigms. For example, Culbertson & Adger (2014) use an ALL paradigm in which participants are trained on phrases in a novel language that consist of nouns modified by either a single adjective, numeral, or demonstrative. The relative order of the modifiers is withheld. At test, when participants have to select a relative order between modifiers, participants tend to infer orders which are homomorphic in that they have the adjective closer to the noun than the demonstrative (Martin, Ratitamkul, Abels, Adger, & Culbertson, 2019; Martin et al., 2020; Martin, Adger, Abels, Kanampiu, & Culbertson, 2024). Similar results have also been obtained in silent gesture studies, where hearing nonsigning participants have to create new ways to convey objects and their properties, for example, texture, numerosity, location relative to the gesturer, using just their hands (Culbertson, Schouwstra, & Kirby, 2020). Participants in these silent gesture experiments show a preference for orders in which gestures conveying properties like “striped” or “spotted” are closer to gestures conveying objects than gestures conveying location. Thus, as for the other category‐specific biases discussed above, while typological tendencies are often stated at the level of syntactic categories (e.g., subjects, nouns, adjectives), evidence from silent gesture experiments points to the possibility that biases originate in semantics may come to influence the conventionalized order in syntax.

This same logic also extends to biases that have effects in other domains. Category‐specific biases have been argued to motivate the prevalence of certain phonological rules, such as the widespread use of vowel harmony—a phonological assimilation rule where vowels within a word change to share specific properties like rounding or place of articulation. The bias for vowel harmony is typically argued to be driven by phonetics, in particular, vowel‐to‐vowel co‐articulation (Ohala, 1994) between individual vowels within a word. This bias potentially leads to a preference for phonological assimilation—that is, vowel harmony rather than disharmony. Recent ALL studies have found that participants show a preference for vowel harmony over disharmony, suggesting that a category‐specific bias which has its origins in phonetics can nevertheless impact phonological typology (Martin & Peperkamp, 2020; Martin et al., 2019).

Interestingly, not all of the category‐specific biases we have exemplified above correspond to obvious typological tendencies at the level of the grammar. For example, there are few languages that seem to have a productive distinction in word order based on event type. So far, it has only been found in Brazilian Sign Language (Napoli, Spence, & de Quadros, 2017), and in Nicaraguan Sign Language (Flaherty, Schouwstra, & Goldin‐Meadow, 2018). This could be the result of the system‐wide bias for regularity mentioned above. This bias can pull against category‐specific preferences that would otherwise favor variation in word order based on specific categorise present in the utterance context. Using one word order consistently reduces variability *across the system*, and a range of experiments have found evidence for such a bias, especially if the task involves learning of linguistic stimuli (Culbertson et al., 2012; Ferdinand, Kirby, & Smith, 2019; Samara, Smith, Brown, & Wonnacott, 2017; Smith et al., 2017; Smith & Wonnacott, 2010). In other words, a system‐wide bias for word order consistency may compete with category‐specific biases that would otherwise favor word order variation. This kind of bias could lead to the loss of patterns arising from category‐specific biases, such as conditioning based on event type which may have been present in earlier stages of language evolution. Successive generations of learners may impose the more systematic use of one word order, with the other variant gradually being lost.

Returning to harmony, here we argue that like the type of basic word order regularity described, it is best considered as the result of a system‐wide bias. While the tendency for languages to be harmonic can be seen both across and within phrase types (Dryer, 1992; Greenberg, 1963; Hawkins, 1990), to evaluate adherence to the harmony bias, a single item containing just one head and a dependent (e.g., a Head‐Dependent phrase) provides insufficient evidence. Additionally, while a single phrase (e.g., a Head‐Dependent1‐Dependent2 phrase) would give evidence of harmony within that phrase, a system can be harmonic without ever exhibiting complex phrase structure. In such cases, the only way to know whether a system is harmonic is by evaluating the structure of phrases *across* the system. In line with this, previous research using ALL to examine the preference for harmonic word order involves providing learners with evidence for harmony only across phrases which by themselves only feature a single dependent. These studies find clear evidence that participants tend to learn harmonic word orders better than nonharmonic ones. For example, studies employing a regularization paradigm, where participants are trained on variable word order, find regularization of variable harmonic systems more readily than nonharmonic ones (Culbertson & Newport, 2015; Culbertson et al., 2020; Culbertson et al., 2012). This shows that alignment of orders across phrases is enough to activate the harmonic bias, and that there may be a system‐wide cognitive bias for consistent order of heads relative to dependents across different phrase types, which contributes to the typological propensity for harmony.

To summarize, we have identified two types of cognitive biases, category‐specific and system‐wide, which have been argued to shape language typology on the basis of experimental evidence. The key diagnostic we propose to distinguish category‐specific from system‐wide biases is whether it suffices to consider a single item or unit to evaluate bias adherence, or whether instead the larger linguistic system (or grammar) in which the unit occurs must be taken into account. Both types of biases can target different levels of abstraction, and the mechanisms of the bias are in principle irrelevant to this distinction, though here we have highlighted several cases in which biases contributing to word order typology may have their origin in meaning rather than syntax.^3^ What is of particular interest in the remainder of this paper is the fact that system‐wide and category‐specific biases can *conflict* with each other.

As outlined above, a potential example of this type of conflict is between a system‐wide bias for consistent basic word order and category‐specific biases for event‐ (verb‐) type conditioning. Similarly, a system‐wide bias for harmony may also be in competition with category‐specific biases. Indeed, there are clear typological exceptions to word order harmony which might reflect this. For example, although noun phrase dependents tend to exhibit harmony, certain dependent types are more likely to stand out as exceptions. In particular, adjectives (“the red house”) are more likely to be postnominal, and genitives (“the child's toy”) are more likely to be prenominal, regardless of the order of other dependents (see Tables 1 and 2, based on spoken language data in WALS Dryer, 2013a, 2013b). The impact that this has on harmony can be seen by looking at the number of spoken languages exhibiting harmonic versus nonharmonic orders of these two elements relative to the noun (see Table 3, based on spoken language data in WALS Dryer, 2013a, 2013b). The nonharmonic order where the genitive precedes the noun and the adjective follows it is just as common as the postnominal harmonic order. The prenominal harmonic order and the nonharmonic order with prenominal adjectives but postnominal genitives are both much less common. A similar pattern is also observed in typological data based on sign languages, where most languages exhibit postnominal ordering for adjectives and prenominal ordering for genitives (Coons, 2022). This deviation from the harmonic pattern suggests that there may be two category‐specific ordering biases—one which leads to a preference for postnominal adjectives, and another which leads to a preference for prenominal genitives—that compete with a system‐wide bias for harmony. Below, we posit that these category‐specific biases themselves may target *semantic* categories, that is, descriptive properties and possessors. While these semantic categories can be expressed using a number of syntactic categories, descriptive properties are often expressed using adjectives and possession is often expressed using genitives, and so the ordering preferences for these types of meanings can come to influence syntactic typology.^4^

**Table 1:** Order of adjectives in relation to nouns in spoken languages

Order	*N*
Noun‐Adjective	879
Adjective‐Noun	373
Other	110

**Table 2:** Order of genitives in relation to nouns in spoken languages

Order	*N*
Noun‐Genitive	468
Genitive‐Noun	685
Other	96

**Table 3:** Order of adjectives and genitives in relation to nouns in spoken languages

Order	Noun‐Adjective	Adjective‐Noun
Noun‐Genitive	342	65
Genitive‐Noun	342	232

### 1.2 Different biases in different contexts

The research reviewed above suggests an intriguing pattern: evidence for system‐wide and category‐specific biases appears in different contexts. For example, most experiments revealing a bias for word order harmony involve participants learning a language system and being asked to reproduce it (Christiansen, 2000; Culbertson et al., 2020; Culbertson et al., 2012). By contrast, in a task where participants have no model/input and have to improvise gestures for object properties—numerosity, texture, objects, and relative location—no preference for harmonic orders is found. Simply put, participants do not tend to gesture all properties before or after the object (Culbertson et al., 2020).^5^ Conversely, it is in precisely these improvisational experimental contexts that category‐specific preferences have been found. The bias for homomorphism was found in studies where participants either improvise in the absence of any conventionalized system, or innovate the relevant part of the system (Culbertson et al., 2020; Martin et al., 2020; Martin et al., 2019). Similarly, preferences for basic word order patterns specific to particular categories of verbs or event types have emerged under these same conditions (Schouwstra & de Swart, 2014; Motamedi et al., 2021).

These findings suggest the possibility that system‐wide and category‐specific biases emerge via distinct mechanisms, or at least, in distinct linguistic contexts that require different amounts of learning versus improvisation. Category‐specific biases may influence behavior in contexts requiring improvisation, when there is no firm language system already in place; system‐wide biases may influence language during learning, where the different parts of the system are all in play. If this is true, it is worth considering whether and how these contexts might relate to stages of language evolution. For example, if category‐specific biases are found most readily when no conventionalized system is in place, then they may have a relatively limited window in which to influence language structure (e.g., early during language emergence, or when a completely novel structure or combination must be improvised in the context of an already established language). If system‐wide biases are found during learning—that is, when learners are storing or retrieving learned patterns, or generalizing to items or contexts that are similar to those—these biases would be less restricted in their influence. Such biases could shape language continuously, exerting pressure anew on languages with each new generation of learners.

However, it is worth noting that some syntactic effects of category‐specific biases appear to be quite robust—like the typological tendency for adjectives to be postnominal, and genitives prenominal. This suggests the possibility that at least some category‐specific biases might also influence learning. In other words, these biases may emerge not only in improvisation, but may also make a linguistic system which aligns with them easier to learn. Few studies have directly investigated this possibility, but recent work has found some evidence that systems in which basic word order aligns with category‐specific biases for event type are indeed easier to learn (Motamedi et al., 2021).

Here, we pursue this question further by investigating the possibility that category‐specific biases affect both improvisation and learning when participants are tasked with creating and learning a language system involving both descriptive and possessive expressions. We do this using the silent gesture paradigm. This paradigm has been argued to tap into participants' preferences with less influence from their native (spoken) language, since it is in a different modality, and does not directly map onto particular syntactic structures in their language. For example, previous silent gesture studies have revealed shared gesture ordering preferences across participants with distinct native language orders, suggesting that gesture order does not recapitulate native language orders (Goldin‐Meadow et al., 2008; Hall et al., 2013). Notably, the fact that gestures do not necessarily map onto specific grammatical categories also means that evidence for ordering preferences in these experiments do not directly link to syntactic typology. As in previous work on silent gesture, we assume that gestures provide a representation of the type of *information* that tends to be conveyed by particular grammatical categories of interest (such as adjectives and genitives). The biases we find in silent gesture indicate preferences for ordering information in certain ways. The assumption underlying our research (following much previous work, e.g., Goldin‐Meadow et al., 2008; Meir et al., 2017; Schouwstra & de Swart, 2014) is that preferences for how information is ordered can come to influence how syntactic structure is conventionalized through continuous application of these biases in the minds of individuals. In our case, since expressions of descriptive properties and possessors can be linked to conventionalized syntactic categories, like adjectives and genitives, we argue that the gesture orders that participants produce in our experiments can shed light on why nominal typology looks the way it does. In Experiment 1, we conduct a silent gesture task where there is no conventionalized linguistic system in place. We provide the first behavioral evidence that, in this context, participants have a preference for descriptive expressions to follow object gestures but for possessive expressions to precede object gestures. In Experiment 2, we then test whether these preferences continue to influence participants' behavior in a silent gesture learning task. Here, the testing materials are identical, but are preceded by a stage in which participants are exposed to evidence for a conventionalized gesture order they must learn.

## 2 Experiment 1

In this experiment, we use a silent gesture perception task to test participants' ordering preferences for possessive and descriptive expressions in the absence of evidence of a wider, conventionalized, linguistic system. As noted above, we take a preference for gestures in which the information conveying the object in an image comes before the information conveying that object's properties as in line with a preference for postnominal descriptive expressions (e.g., adjectives). We take a preference for gestures in which the information conveying the object in an image comes after the information conveying that object's owner as an indication in line with a preference for prenominal possessive expressions (e.g., genitives). We use the terms descriptive and possessive throughout, and we use the terms pre‐ and postnominal for convenience to refer to the entity/object gesture.

Our first experiment examining these proposed ordering preferences uses a between‐subjects design, manipulating meaning type: either descriptive or possessive. Following the method used in Motamedi et al. (2021), participants were given a single trial in which they were asked to choose between two gesture videos. Here, one video uses a prenominal gesture order for the target meaning and the other a postnominal gesture order for the target meaning.^6^ We predict that participants will exhibit a preference for postnominal order in the descriptive condition, but prenominal order in the possessive condition. If these preferences are found, this would support the notion that the ordering of the expressions of these meaning types is subject to category‐specific biases.

### 2.1 Methods

#### 2.1.1 Materials

**Fig. 1:**
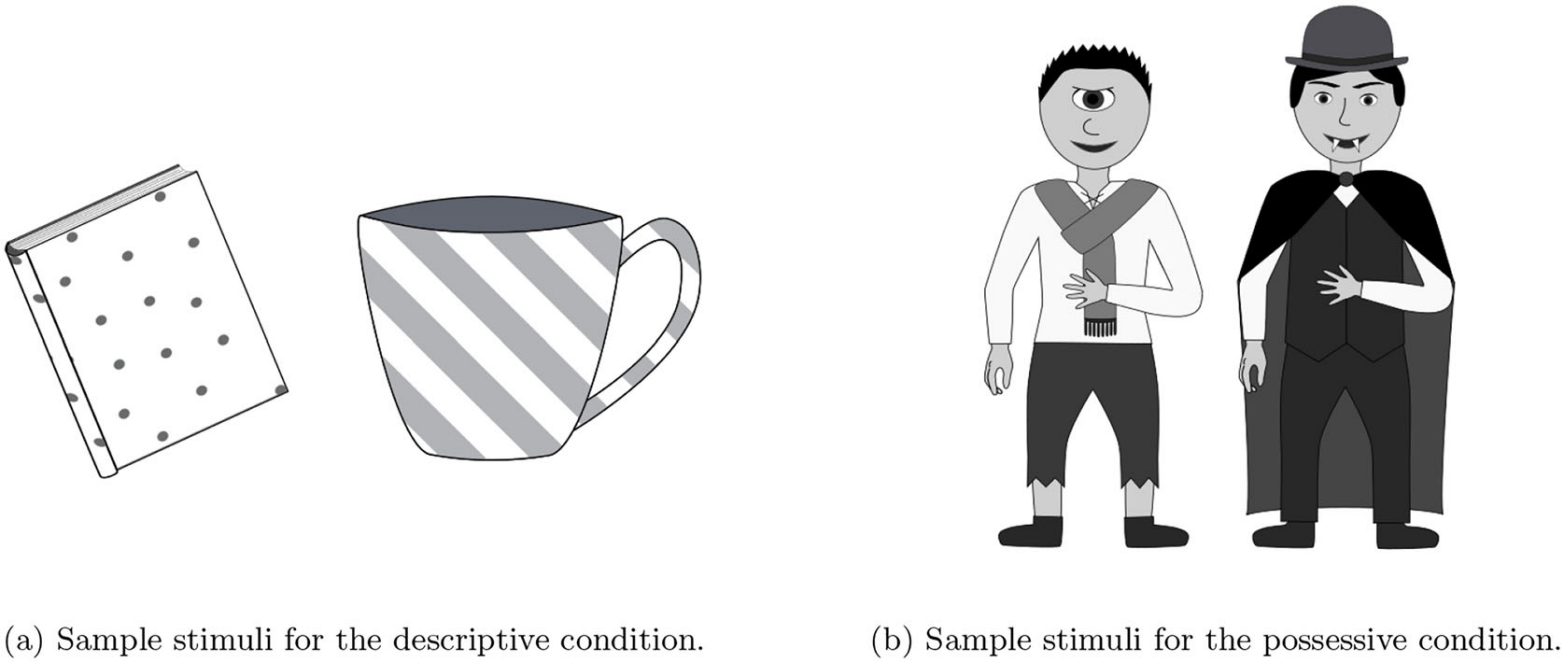
These samples show a subset of the total possible set of stimuli images.

The experiment was developed using the JavaScript library jsPsych (de Leeuw, 2015) and ran in participants' web browsers. Participants saw a collection of grayscale digital drawings showing either instances of item ownership (possessive condition, e.g., “vampire's hat”) or items with different patterns (descriptive condition, e.g., “striped cup”). The set of images consisted of every possible combination of the two possessives (possessors) “vampire” and “cyclops,” the two descriptive features “spotted” and “striped” as well as four nouns “hat,” “scarf,” “cup,” and “book.” The images were created in Inkscape and, in total, there were 16 possible images each representing a different meaning (see Fig. 1 for sample images from each condition). We follow previous research (Culbertson et al., 2020; Jaffan, Klassen, Yang, & Heller, 2020), which has found some evidence supporting a preference for postnominal descriptive expressions when using inanimate objects in the image stimuli for the descriptive condition. For the possessive condition, we instead use animate (human‐like) entities as possessors. We made this decision because this experiment is the first test of the preference for prenominal possessive expressions, and these are the types of stimuli we would expect to be most likely to elicit the preference. This is because a general preference to have animate or human entities first has been documented in previous research (e.g., as an alternative formulation of the subject‐first bias, as well as in other psycholinguistic tasks Meir et al., 2017; Prat‐Sala & Branigan, 2000). If the preference for prenominal possessive expressions is observed with these types of possessors, future work could explore whether it is also present with inanimate possessors.^7^

For each image, there were two gesture videos, making a total of 32 videos. The videos showed a model gesturer producing two gestures in sequence, one representing the head noun and one representing one of the two meaning types, either a descriptive or possessive meaning. The videos differed only in the order of these two gestures—in one, the head noun was the first gesture, in the other, it was the last. Each phrase component was denoted using a gesture made with both hands and the videos ended with both hands in a neutral position. The videos were all 4389 ms long and matched so that the beginning and end of each component gesture was synchronized across each pair of videos.

#### 2.1.2 Procedure

**Fig. 2:**
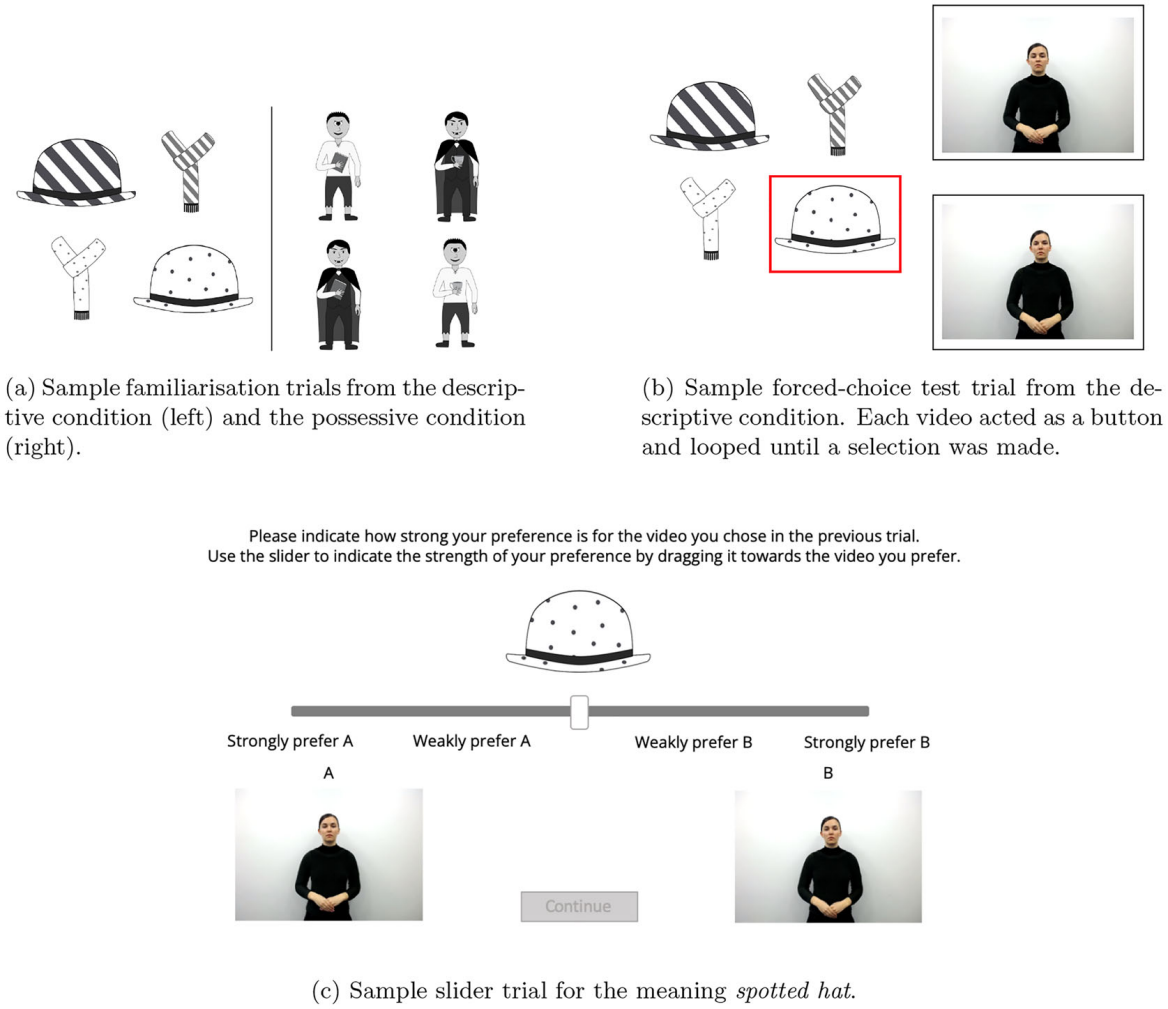
Illustrative examples of the main trials in Experiment 1.

Participants were randomly assigned to either the descriptive condition or the possessive condition. They were instructed that the study was about “how to describe items in a sign language” if they were in the descriptive condition, or “how you express ownership in a sign language,” if they were in the possessive condition. Prior to the main testing trial, participants were shown a sample 2x2 grid of images containing the kinds of images that they would be shown in the test trial and these images exhibited contrasts along both the object and meaning type dimensions (see Fig. 2a).

The instructions for this familiarization trial necessarily included reference to an “item” and either a “pattern” or “owner.” The order in which the noun referent “item” and the two meaning type referents (“pattern” and “owner”) appeared in the instructions was randomized between participants. This was done to avoid the possibility that the order between these elements in the instructions biased participants in favor of a certain gesture order in the test trial. The alternative instructions that participants saw included either “Across the images both the patterns and the items vary” or “Across the images both the items and the patterns vary.” in the descriptive condition and “Across the images both the owners and the items vary” or “Across the images both the items and the owners vary” in the possessive condition. We examine the effect of this ordering as part of our preregistered exploratory analysis in the Results section.

After this pre‐test trial, participants were instructed that they would see the same kind of 2x2 grid but with one image highlighted in red. They were told that two videos would appear next to the image grid and that these represented two ways to express “ownership of the item” in the highlighted image (in the possessive condition) or two ways that the highlighted “item could be described” (in the descriptive condition) in a made up sign language. Their task was to choose the gesture video which they thought best conveyed the meaning of the highlighted image. The images remained on the screen, with the videos looping next to them, until participants chose one gesture video by clicking on it (see Fig. 2b for an example).

Following this single test trial, a second trial asked participants to drag the point of a slider to indicate how strong their preference was for the gesture order they chose in the previous forced‐choice trial. The target image from the forced‐choice trial was displayed above the slider and the two videos looped on either side of the slider and were labeled “A” (for the left video) and “B” (for the right video). The location of the prenominal and postnominal video was randomized per participant. To submit a response, participants had to drag the slider point from the middle toward one of the videos. The slider was marked with “weakly prefer video A/B” and “strongly prefer video A/B” on either side of the mid‐point (see Fig. 2c for example of slider trial). Following this, participants were shown the video they had chosen in the forced‐choice trial and were asked to translate the meaning of the gesture video into English by typing in a response. ^8^ Finally, participants responded to two short demographics questions. One asking them if they knew a sign language (used for exclusions) and another asking them to note which spoken languages they knew and at what level of proficiency (on a scale of 1–10, where 10 was native‐like proficiency).^9^

#### 2.1.3 Participants

A total of 384 participants were recruited via the online crowdsourcing platform Prolific. Using the built‐in Prolific prescreening options, we restricted participation to those who reported English as their first language, had at least a 95% previous task approval rate, and had not completed any of our previous experiments or pilots. Participants were paid the equivalent of £8.91 per hour. We excluded eight participants who stated that they were proficient in a sign language. A further 56 participants were excluded as they responded too quickly to the forced‐choice trial (< 9678 ms, combined time for both videos, meaning they had not watched both videos before making their choice; *N* = 18), did not indicate a preference for the same gesture video across both the forced‐choice and slider trial (*N* = 24), or both (*N* = 14). After these (preregistered) exclusions, there were 160 participants in each condition.

### 2.2 Coding

The forced‐choice trial responses were coded using a binary variable, *predicted order*, with 1 for the predicted order (prenominal order in the possessive condition, postnominal in the descriptive condition) and 0 for the alternative order (postnominal in the possessive condition and prenominal in the descriptive condition). The slider trial responses were transformed to account for the fact that values close to 0 represented a strong preference for the video on the left, and a value very close to 100 represented a preference for the video on the right. To make these preferences comparable, independently of video placement, all values under 50 were converted to their corresponding value above 50 (e.g., 2 becoming 98).

### 2.3 Results

Based on the typological data, we made two main predictions for Experiment 1: (i) participants will prefer the postnominal gesture order when the gestures expressed a descriptive meaning, (ii) participants will prefer the prenominal gesture order when these expressed a possessive meaning. We also made an additional prediction based on the typological data, where the asymmetry in the prenominal versus postnominal order for adjectives and genitives is such that the postnominal adjective preference appears (numerically) stronger than the prenominal genitive preference (Dryer, 2013a, 2013b). Therefore, we predicted: (iii) the postnominal preference for descriptive meanings will be stronger than the prenominal preference for possessive meanings.

#### 2.3.1 Main analysis

**Fig. 3:**
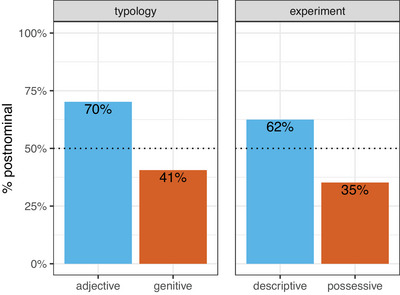
Proportion of postnominal orders per dependent and meaning type based on the typological data (left facet) and selections of participants in the forced‐choice trials (right facet). In both the typological and the experimental data, postnominal order is preferred for adjectives/descriptive expressions and prenominal order is preferred for genitives/possessive expressions.

To evaluate our first two predictions, we first examined the extent to which participants chose the predicted order in forced‐choice trials across the two conditions. As shown in Fig. 3, participants' choices closely match what is observed in the typological data for spoken languages (Dryer, 2013a, 2013b). According to our preregistered analysis plan, the data were analyzed using mixed effects logistic regression models implemented using the lme4 package (Bates, Mächler, Bolker, & Walker, 2015) in R (R Core Team, 2013). Results from two intercept‐only models (one per condition), with *predicted order* as the outcome variable, indicated that participants chose the predicted order for their respective conditions at rates significantly above chance (possessive condition: β = 0.56, *SE* = 0.16, *z* = 3.43, *p* < .001, descriptive condition: β = 0.51, *SE* = 0.16, *z* = 3.02, *p* < .01).^10^ These results support our first two predictions.^11^

**Fig. 4:**
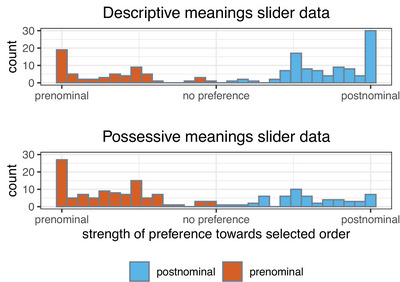
Number of participants per condition that indicated different strengths of preference in favor of the order chosen in the forced‐choice phase of the experiment. Bars far to the left indicate a strong prenominal preference and bars far to the right indicate a strong postnominal preference. More central bars indicate weaker preferences. The patterns of preferences are broadly similar across both conditions but overall they were slightly higher in the descriptive condition.

We can also test whether participants who chose the predicted order on the forced‐choice trial also showed a stronger preference for these orders (i.e., gave them a higher rating) in the slider task, compared to those who chose the alternative order. Fig. 4 shows the number of participants in each condition who indicated a given preference strength on the slider trials. To analyze these data, we ran two linear models, one for each condition. The outcome variable was the transformed *rating* values, with a fixed effect of *predicted order*. While Fig. 4 suggests that, when plotting counts of participants who chose a specific preference strength, more participants gave the highest rating to predicted orders in each condition, neither model reached significance (possessive condition: β = 3.89, *SE* = 2.14, *t* = 1.81, *p* = .071, descriptive condition: β = 1.06, *SE* = 2.07, *t* = 0.51, *p* = .71).^12^ To summarize, participants were more likely to choose the predicted order on forced‐choice trials. However, there is no evidence that preference ratings in the slider task were stronger for participants who chose the predicted order, compared to those who did not.

To evaluate our third prediction, that the preference for postnominal descriptive expressions would be stronger than the preference for prenominal possessive expressions, we again analyzed both the forced‐choice and slider data. We analyzed the forced‐choice data using a logistic regression model with *predicted order* as the outcome variable and *condition* as a fixed effect (the descriptive condition acted as baseline). This model revealed no difference between the descriptive and possessive conditions (β = 0.05, *SE* = 0.23, *z* = 0.23, *p* = .72).

We also evaluated this prediction for the slider data, using a linear model with transformed *rating* as the outcome variable, and *predicted order*, *condition* (the descriptive condition acted as baseline), and their interaction as fixed effects. The results revealed a significant negative coefficient for condition (β = −4.93, *SE* = 2.36, *t* = −2.09, *p* = .04), indicating that preference ratings were, overall, slightly lower in the possessive condition than in the descriptive condition. However, no significant interaction was found. To summarise, neither the forced‐choice data nor the slider data provide evidence for a stronger preference for the predicted order in the descriptive condition compared to the possessive condition.

#### 2.3.2 Exploratory analysis

In addition to testing our three preregistered main predictions, we also conducted an exploratory analysis. This was done to rule out the possibility that the order of words in the instructions for the familiarization trial influenced the gesture order participants chose in the forced‐choice task (see Section 2.1.2 for details about word order in instructions). We ran two logistic regression models, one per condition, with the binary outcome variables *prenominal* (possessive condition) and *postnominal* (descriptive condition) with the two‐level fixed effect of *instruction order* (prenominal or postnominal; postnominal as baseline). Neither of these models reached significance (possessive model: β = −0.25, *SE* = 0.33, *z* = −0.75, *p* = .46, descriptive model: β = −0.12, *SE* = 0.33, *z* = −0.37, *p* = .71); therefore, there is no evidence that the order of the words in the instructions determined the choice participants made in the experimental forced‐choice trial.

### 2.4 Experiment 1: Discussion

In Experiment 1, participants were tasked with choosing (and rating) their preference for a gesture order that expressed either a descriptive or possessive meaning. They did this in the absence of any evidence about the wider linguistic system, and for only a single exemplar meaning. The results confirm our main predictions: the orders participants preferred for descriptive and possessive meanings in a silent gesture preference task align with the most common orders we see for adjectives and genitives in both spoken and sign language typology (Coons, 2022; Dryer, 2013a, 2013b). This suggests that the typology may reflect category‐specific biases for the types of meanings often expressed by these two dependent types. These biases may also explain the absence of harmony between these two dependent types: category‐specific ordering preferences may work to keep these dependents split across the head noun.

While we have found clear evidence for these two category‐specific biases, we failed to find any evidence that one was stronger than the other. There was no overall difference in the likelihood of choosing the predicted order across our two conditions, and the preference ratings were not stronger for postnominal descriptive meanings compared to prenominal possessive meanings.

There are a number of potential explanations for this. First, it could be that this particular asymmetry simply reflects some other mechanism—like accidental facts about language history—since of course simply counting the numbers of languages that have one pattern versus another does not control for genetic or areal relationships among languages. However, it is also possible that the prenominal possessive preference in our experiment was particularly strong due to the fact that we used only animate/human‐like possessors (as noted above). It is also possible that the lack of difference in the strength of participants' preferences could reflect some influence from their native language, despite somewhat limited evidence for the influence of participants' spoken language on gesture from previous studies (Culbertson et al., 2020; Goldin‐Meadow et al., 2008; Hall et al., 2013). English has variation in the order of genitives, and even some variation in the order of adjectives; however, for the types of meaning used in this experiment, prenominal order is preferred for both (e.g., *vampire's hat*, *spotted hat*). A prenominal preference coming from English experience might, therefore, strengthen the more general preference for prenominal possessive expression, but weaken the more general preference for postnominal descriptive expression. This would reduce the difference between the two meaning types in the experiment, leading to a failure to exhibit the predicted asymmetry. Yet, if influence from English syntax is active in our task, it is striking that we still find a preference for descriptive meanings to be expressed postnominally, given that English tends to express such meanings prenominally.

Finally, it is worth mentioning that the strength of the prenominal possessive preference might be driven by a modality‐specific effect for this particular type of item. Previous research has shown that some ordering preferences observed in silent gesture studies may be influenced by modality‐specific constraints (Napoli & Sutton‐Spence, 2014; Struhl, Salinas, Lim, Fedorenko, & Gibson, 2017). In our case, the gestures denoting the possessor used in this experiment, the body of the gesturer is used to inhabit the role of the animate referent by representing either the vampire's body or the cyclops' body. Studies on sign languages and silent gesture have found that signs which make use of the body in this way are often linearized earlier in production, which, in this case, would create a stronger prenominal genitive preference (Meir et al., 2017). This type of modality‐specific effect could explain why the typological data from sign languages show the same directionality of ordering preferences (Coons, 2022), but a (numerically) stronger preference for prenominal genitives compared to postnominal adjectives. If this modality‐specific effect is at work here, it might strengthen the prenominal possessive preference and, therefore, obscuring the difference in bias strength that we would predict based on the spoken language typological data for adjectives and genitives. Independently of any potential boost to the prenominal possessive preference that the manual modality may afford, the typological data based on spoken languages suggest that the prenominal genitive preference also extends to the spoken modality where animate entities are denoted using vocal cues. Therefore, it is unlikely that the overall prenominal possessive preference we observe in this experiment is purely due to modality‐specific effects.

Regardless of the lack of difference in the strength of the two preferences, we have found here clear evidence for category‐specific preferences influencing order in the absence of a linguistic system. In this task, where participants received no evidence of a conventionalized linguistic system, gesture orders in which the object gesture precedes the object description, but follows the possessor, were clearly preferred. Under the assumption that preferences for ordering information influence conventionalized syntax, these preferences align with the postnominal adjective and prenominal genitive tendency found in typology. In the next experiment, we ask whether these same orders are also easier to *learn*.

## 3 Experiment 2

Experiment 2 tested whether the ordering preferences observed in Experiment 1 also influence how participants learn gesture order in a novel miniature system. In principle, one could simply ask whether fixed ordering systems that use one of the preferred orders (e.g., corresponding to postnominal descriptive expressions or prenominal possessive expressions) are easier to learn. However, there is evidence that in simple artificial language learning experiments, adults very easily learn and reproduce even very rare noun phrase orders (Culbertson & Newport, 2017). In other words, we expect preferences to be relatively subtle. Therefore, we follow others in using a regularization design, in which participants are exposed to a system with some unpredictable variation. Specifically here, multiple orders are possible (e.g., both prenominal and postnominal descriptive expressions), but one order is more common. This design capitalizes on the fact that learners tend to regularize rather than reproduce unpredictable variation (Ferdinand et al., 2019; Hudson Kam & Newport, 2009; Smith & Wonnacott, 2010), but regularization is more likely when the majority order is preferred (Culbertson & Newport, 2015; Culbertson et al., 2012).

We test whether participants are more likely to learn and regularize the majority order they are exposed to when this order aligns with the category‐specific biases identified in Experiment 1—that is, gesture orders aligning with postnominal descriptive expressions or prenominal possessive expressions. Finding evidence that the preferences from Experiment 1 continue to influence participants' behavior when they are tasked with learning an existing language system could help explain the pattern we see in the typological data, namely, that the two dependent types that often instantiate these meanings, adjectives and genitives, resist harmony by appearing on different sides of the head noun. More generally, if category‐specific preferences are active not only during language emergence, but also during language learning, then they have more opportunity to influence language structure.

Experiment 2 was a between‐subjects silent gesture design, similar to Motamedi, Wolters, Naegeli, Schouwstra, and Kirby (2021). There were four conditions, created by crossing the two variables of interest, namely, *meaning type* (either descriptive or possessive), and what we will call the *naturalness* of the majority order that participants were trained on (either natural or unnatural), where natural orders were those which aligned with the category‐specific preferences found in Experiment 1. The conditions were called “natural descriptive,” “unnatural descriptive,” “natural possessive,” and “unnatural possessive.” Participants were first trained on example gestures in each condition, as shown in Table 4. They then completed the same type of forced‐choice perception task as in Experiment 1.^13^

**Table 4:** Percentage of prenominal and postnominal gesture orders in input per condition

Condition	Prenominal	Postnominal
Natural descriptive	25%	75%
Unnatural descriptive	75%	25%
Natural possessive	75%	25%
Unnatural possessive	25%	75%

### 3.1 Methods

#### 3.1.1 Materials

The second experiment was built using the same technical tools as Experiment 1 and used the same stimuli images and gesture videos. Participants were randomly assigned to one of the four conditions and a pseudo‐randomized stimuli set containing two target meanings and associated distractors. The stimuli set consisted of two nouns, one from the set of “worn” items (i.e., “hat” or “scarf”) and one from the set of “held” items (i.e., “cup” and “book”). Each of these two nouns was then paired with one of the two meaning types associated with the condition. For example, a stimulus set for a participant in one of the descriptive conditions might consist of “striped hat” and “spotted cup.” The other three images in the 2x2 grid used in training and testing trials were chosen in the same way as in Experiment 1.

#### 3.1.2 Procedure

Participants were instructed that they were going to learn how to express “ownership of an item” in a made‐up sign language (possessive conditions) or that they would learn how to “describe an item” (descriptive conditions). Prior to the training phase, participants were exposed to the same kind of familiarization trial as in Experiment 1. Following this, the main training phase took place and participants were told that a similar 2x2 grid would appear, but with one meaning highlighted, and that below the images, they would see a video of a person using gestures to convey the meaning in the highlighted image. All they had to do was sit back and watch carefully as each of the two target images were displayed with their corresponding gesture videos eight times each. Six of eight times the image would be described in the majority gesture order for that condition, and twice in the minority order. The training phase trials progressed automatically.

After this, participants were tested on what they had learned. They saw the same kind of image grid as in the training phase, but both possible gesture videos that corresponded to the target image were displayed under the images. These two videos looped simultaneously until participants chose one of them by clicking on it. Participants were instructed to “click on the corresponding gesture video” like they had seen for those meanings during training. The testing phase had the same number of trials as the training phase (16) and participants saw both target meanings eight times and clicked a centered “Next” button to proceed between trials. The location of the gesture videos (left or right) was randomized per trial per participant.

After the training and testing phases, participants were presented with translation trials, similar to Experiment 1 but twice, once for each target meaning. The gesture order they were prompted with for each target meaning was pseudo‐randomized so that one meaning appeared with a prenominal gesture order and one meaning with the postnominal one. Finally, participants answered the same demographics questions as in Experiment 1. ^14^

#### 3.1.3 Participants

A total of 215 participants were recruited via the online crowdsourcing platform Prolific. We employed the same prescreening requirements as in Experiment 1. Participants were paid the equivalent of £9.50 per hour. We excluded six participants who stated that they were proficient in a sign language. A further five participants were excluded as they did not provide coherent responses to the translation and/or demographics questions (e.g., only included a random sequence of letters). Finally, one participant was excluded for pressing the same button over 90% of the time and data from three participants were lost due to technical issues during the experiment. After these preregistered exclusions, there were 47 participants in the natural descriptive condition, 50 in the unnatural descriptive condition, 50 in the natural possessive condition, and 53 in the unnatural possessive condition.

### 3.2 Results

We had three main predictions for Experiment 2: (i) participants would show evidence of having learned the gesture orders they were trained on, by either reproducing and/or regularizing the majority variant from their training. This prediction is a check to be sure that participants learn from the training data. Second (ii), we predicted that participants' learning behavior would be modulated by the naturalness of the majority variant in their condition: participants in the natural conditions were predicted to regularize more readily than participants in the unnatural conditions. Third (iii), we also predicted that participants would show an overall preference for natural orders by selecting more natural orders than predicted by chance across all conditions, regardless of the majority order. Finally, as in Experiment 1, we made the additional prediction that the naturalness preference would be stronger for descriptive than possessive meanings (i.e., an interaction between naturalness and meaning type).

#### 3.2.1 Learning

**Fig. 5:**
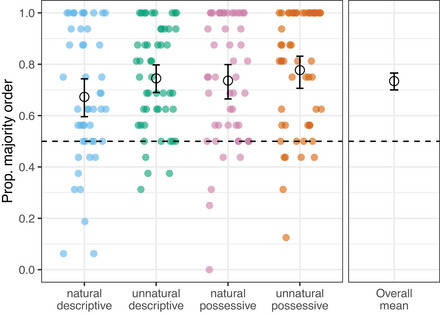
Overall mean (right panel), condition means (black circles, left panel), and individual participant proportions (colored dots) of test trials where participants chose the majority input order for each condition. Error bars represent bootstrapped 95% CIs around the means. Dashed line shows chance‐level performance. Participants tended to produce more of the majority orders from their training than is expected by chance, and there was no difference between conditions.

We first analyze whether participants generally learned the orders they were trained on, and whether this was modulated by condition as predicted. Fig. 5 shows proportion choice of the majority orders in each condition (with right‐hand panel collapsing across conditions) in the testing phase. We ran a mixed effects logistic regression model with *majority order* as the binary outcome variable (1 when participants' choice matched the majority order they were trained on, and 0 when it did not), and fixed effects for *majority natural* (either natural or unnatural) and *meaning type* (either descriptive or possessive) as well as their interaction. Both fixed effects were deviation‐coded (possessive = 0.5 and descriptive = −0.5, natural = 0.5 and unnatural = −0.5). The models also included a random slope for participants. The model had a significant positive intercept (β = 1.51, *SE* = 0.13, *z* = 11.62, *p* < .001) showing that, on average, participants across all conditions choose the majority order at a rate above chance. This confirms our first prediction, that participants generally learned from the gestures they were trained on. Model comparison using a likelihood ratio test revealed that the null model (reported above) was the best fit for the data, and that including *majority natural*, *meaning type*, or their interaction did not improve model fit ($\chi^{2}=2.01$, *p* = .16; $\chi^{2}=2.91$, *p* = .09; $\chi^{2}=0.005$, *p* = .94). This indicates that there was no reliable difference in the likelihood of selecting majority orders for participants in the two natural conditions compared to the two unnatural conditions, nor for participants exposed to descriptive or possessive meanings.

#### 3.2.2 Regularization

**Fig. 6:**
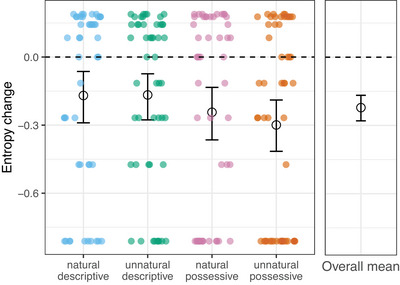
Overall mean (right panel), condition means (black circles, left panel), and individual participant values (colored dots) for changes in entropy between input and output. Error bars represent bootstrapped 95% CIs around the means. The dotted line at 0 represents no change in entropy. Participant dots above this line signify an increase in entropy between input and output. There is an overall tendency toward greater regularity in participants' outputs, and there was no difference between conditions in terms of regularization behavior.

The above analysis confirms that participants across all conditions were able to learn the order they were trained on, since they produced the majority order at a rate significantly above chance. However, this experiment was primarily designed to test participants' regularization behavior across conditions. Here, we quantify an increase in regularity in the system as a decrease in overall entropy between the input (training) and output (forced‐choice selections) following, for example, Ferdinand et al. (2019), Motamedi et al. (2021), and Samara et al. (2017). The entropy (*H*) of a system is defined in this context as:

$$H\left(V\right)=-\sum_{v_{i}\varepsilon V}p\left(v_{i}\right)\mathrm{lo}g_{2}p\left(v_{i}\right)$$

where (*V*) refers to the two possible gesture variants (prenominal and postnominal). All conditions had an input entropy of approximately 0.711, with a maximum possible entropy value of 1 (indicating that the output data shows an exactly 50/50 split between the two orders), and a minimum of 0 (indicating that the output data contains only a single order). The change in entropy was calculated by taking the output entropy of each participant, based on participants' selections in the testing‐phase, and subtracting the input entropy value for their condition. Fig. 6 shows the mean entropy change in each condition (and collapsing across conditions). To evaluate if these changes are reliably greater than zero, we calculated bootstrapped confidence intervals around the mean entropy changes for each condition.^15^ These were generated using the “boot” package in R (Canty & Ripley, 2021) and based on 10,000 samples. These results were further supported by simulating 10,000 runs of the experiment with the probability of simulated participants choosing prenominal or postnominal order set to the input proportions during training (i.e., 0.75 for the majority order and 0.25 for the minority order). Z‐scores were calculated based on the overall mean change in entropy between the observed experimental mean and the overall simulation mean. Similarly, individual z‐scores were calculated for the mean change in entropy for each condition, compared to the corresponding simulation means. These analyses all indicate a reliable negative change between input and output entropy in each individual condition, and overall across conditions (see Table 5 for z‐scores based on simulations). Importantly, CIs around differences in experimental means between conditions reveal no reliable differences between conditions in terms of regularization behavior (see Table 6).

**Table 5:** Experimental means, simulated means, and resulting Z‐scores for change in entropy

Condition	Exp. mean	Sim. mean	z‐score
Overall	−0.221	−0.047	−13.13
Natural descriptive	−0.169	−0.047	−4.59
Unnatural descriptive	−0.167	−0.047	−4.53
Natural possessive	−0.243	−0.047	−7.44
Unnatural possessive	−0.299	−0.047	−9.51

*Note*. Z‐scores show that all experimental means are reliably different from the simulated means indicating that entropy dropped significantly in all conditions.

**Table 6:** Comparison of mean entropy change per condition

Condition	${\bar{x}}_{a}$ – ${\bar{x}}_{b}$	Lower CI	Upper CI
Natural descriptive – Unnatural descriptive	−0.003	−0.15	0.15
Natural descriptive – Natural possessive	0.07	−0.09	0.24
Natural descriptive – Unnatural possessive	0.13	−0.03	0.29
Unnatural descriptive – Natural possessive	0.08	−0.08	0.23
Unnatural descriptive – Unnatural possessive	0.13	−0.02	0.29
Natural possessive – Unnatural possessive	0.06	−0.10	0.22

*Notes*. Includes 95% bootstrapped CIs around each mean.

All intervals cross 0, showing no reliable differences between conditions.

#### 3.2.3 Naturalness

**Fig. 7:**
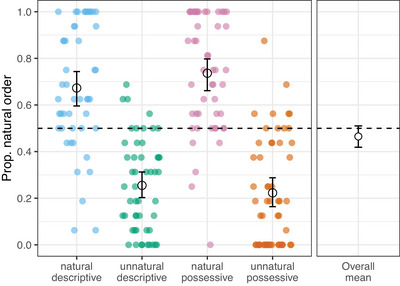
Overall mean (right panel), condition means (black circles, left panel), and individual participant values (colored dots, left panel) for test trials where participants chose the natural predicted natural order for the meaning type they were trained on. Error bars represent bootstrapped 95% CIs around the means. The dashed line at 0.5 shows chance‐level performance. Overall, participants did not tend to produce significantly more natural orders.

Crucially, in addition to our predictions about learning and regularization, we also predicted that if category‐specific biases are active during learning, then natural orders (postnominal descriptive expressions and prenominal possessive expressions) would be chosen more than unnatural orders. Fig. 7 shows the proportion of natural orders chosen by participants in each condition (right‐hand panel collapsing across conditions). We ran a logistic mixed effects model on the binary outcome variable *natural order* (1 if the choice matched the predicted natural order, 0 otherwise). The rest of the model structure was identical to the one used to analyze learning behavior above and thus included fixed effects for *majority natural* (either natural or unnatural) and *meaning type* (either descriptive or possessive) as well as their interaction and a random slope for participants. The intercept term for the model was not significant (β = −0.24, *SE* = 0.17, *z* = −1.36, *p*  = .18), indicating no overall preference for natural orders. A likelihood ratio test revealed that including the fixed effect of *majority natural* improved model fit compared to the null model ($\chi^{2}=115.07$, *p* < .001). There was a significant positive effect of majority naturalness (β = 3.01, *SE* = 0.26, *z* = 11.64, *p* < .001), showing that participants in the natural descriptive and natural possessive conditions were more likely to select the natural order, compared to the grand mean. This is as expected since these were the orders participants were trained on. Including *meaning type* or the interaction between *meaning type* and *majority natural* did not improve model fit ($\chi^{2}=0.001$, *p* = .97; $\chi^{2}=6.12$, *p* = .11).

#### 3.2.4 Mutual information (exploratory)

As is evident from Fig. 6, there are a number of participants whose output in the testing phase shows a higher entropy than their training data. We ran an exploratory analysis (not included in our preregistration) to see whether these participants might have had a different strategy for reducing unpredictable variation, which did not involve regularization as we defined it above. For example, another way in which a system can become more consistent is by reducing the variants (orders) used for a particular meaning/lexical item (Samara et al., 2017; Smith et al., 2017; Smith & Wonnacott, 2010). As participants were exposed to only two target meanings in Experiment 2, they might have conditioned the use of the two gesture orders on these two meanings. This strategy would result in an increase in overall entropy—as participants would use more variable orders across the whole system—but a decrease in variability for a specific meaning. To capture this type of lexically conditioned ordering, and disentangle it from overall entropy, we used a measure of *Mutual Information* of gesture order choice and meaning (lexical item). Mutual information (MI) is computed as:^16^:

MI=overallentropy−conditionalentropybasedonmeaning

**Fig. 8:**
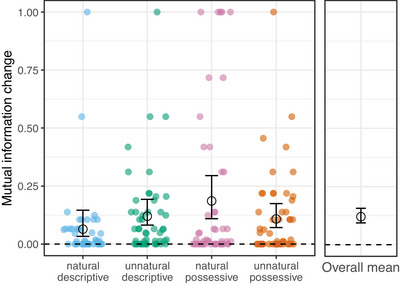
Overall mean (right panel), condition means (black circles, left panel), and individual participant values (colored dots, left panel) for changes in mutual information. Error bars represent bootstrapped 95% CIs around the means. The dashed line at 0 represents no change in MI between training and output. Space above the dashed line represents space of possible MI values. All measures are reliably different from 0, except the change in MI in the natural descriptive condition.

MI of 1 would indicate that participants perfectly condition the two gesture orders on the two meanings they are exposed to, whereas MI of 0 would indicate that participants do not make use of this strategy and that, instead, the variability within each meaning reflects the variability of the system as a whole. The overall mean change in MI across all conditions is 0.12, although there is some variability between individual condition means (see Fig. 8). Based on z‐scores calculated between experimental and simulation means of change in mutual information, the increase in MI is consistent for all condition means except the natural descriptive condition (see Table 7). These results, in combination with the two entropy measures, show that some participants reduced unpredictable variation by using one gesture order more consistently across the whole system, whereas others maintained or even increased overall variability but made this variability predictable based on meaning.

**Table 7:** Experimental means, simulated means, and resulting Z‐scores for change in mutual information

Condition	Exp. mean	Sim. Mean	z‐score
Overall	0.120	0.055	12.27
Natural descriptive	0.064	0.055	0.74
Unnatural descriptive	0.120	0.055	6.18
Natural possessive	0.186	0.055	12.41
Unnatural possessive	0.109	0.055	5.03

*Note*. All experimental means are reliably different from the simulated means, except for the natural descriptive condition.

### 3.3 Experiment 2: Discussion

In Experiment 2, participants learned a variable system of word order for possessive and descriptive meanings where we manipulated whether the input data they were trained on mainly consisted of natural or unnatural orders. Natural orders were gesture sequences where the possessive and descriptive expressions aligned with how conventionalized syntactic systems tend to order nominal dependents, that is, postnominal order of adjectives and prenominal order of genitives. We measured how well participants learned the system they were trained on, the extent to which they regularized the use of one gesture order, and more importantly, their use of natural orders. The results showed that participants learned and regularized the gesture systems they were trained on. However, contrary to our prediction, participants' learning and regularization behavior was not modulated by the naturalness of the majority variant that they were trained on. Participants in the natural conditions did not learn the language systems more accurately, nor did they regularize the majority variant more readily than participants in the unnatural conditions. Similarly, we did not find evidence of any general preference for natural orders across conditions: participants were overall just as likely to choose natural and unnatural orders. Finally, as in Experiment 1, we found no evidence that a preference for naturalness targeted descriptive expressions more than possessive expressions, as would be predicted based on the typological data for noun phrase dependents: the interaction between naturalness and dependent type was not significant.

These results thus do not support the hypothesis that category‐specific biases affect linguistic behavior during the learning of an ordering system, at least in the case of these two meaning types. However, the reliable reduction in entropy across all conditions, combined with the reliable increase in mutual information in three of the four experimental conditions, adds to the body of literature showing that learners are biased against unpredictable variation (Hudson Kam & Newport, 2009; Samara et al., 2017; Smith et al., 2017; Smith & Wonnacott, 2010). Rather than reproducing the unconditioned variation from training, participants tended to overextend the use of one order at test (regularization) or condition the use of the two orders on some lexical aspect of the input.

### 3.4 General discussion

Previous experimental work has found evidence suggesting that cognitive biases, which are active at the level of the individual, may help to explain typological patterns (e.g., Culbertson et al., 2012; Finley, 2018; Martin et al., 2020). For example, there is a typological trend for harmonic word order in the noun phrase (e.g., consistent ordering of dependents before or after nouns), and participants in artificial language learning experiments prefer exactly these orders. In this study, we targeted *an exception* to the typological trend toward harmonic word order in the noun phrase: a nonharmonic order of adjectives and genitives, with adjectives after the noun and genitives before, is just as common as the two harmonic patterns. We sought to explore whether this pattern might be caused by conflicting category‐specific biases influencing individuals ordering preferences. More specifically, we tested the hypothesis that these biases target the order of meanings expressing descriptive and possessive meanings, respectively. Previous experimental work has mainly found that category‐specific biases tend to be active in contexts where participants have little or limited evidence for a wider language system (Culbertson et al., 2020; Martin et al., 2020; Schouwstra & de Swart, 2014), whereas more system‐wide biases—like harmony—are active when these language systems are in place and participants are tasked with learning them (Culbertson et al., 2012; Samara et al., 2017; Smith & Wonnacott, 2010). The experiments in this study investigate both of these contexts. If category‐specific biases are active during both during improvisation *and* learning, this would provide more opportunity for these biases to influence typological structure and, potentially, compete with the system‐wide bias for harmony.

The experiments reported here were thus designed to investigate two main questions. First, we tested whether category‐specific ordering preferences for expressions of descriptive and possessive meanings influence behavior in the absence of a linguistic system—that is, in a task more similar to an improvisation or language create scenario. Second, we tested whether these preferences would influence learning and regularization of a (miniature) gestural linguistic system. Experiment 1 showed that participants have clear preferences when asked to select a gesture order expressing either a descriptive or a possessive meaning without any wider linguistic structure or system being provided to them. Participants in the descriptive condition tended to select postnominal orders for descriptive meanings, whereas participants in the possessive condition tended to select prenominal orders for possessive meanings. These preferences align with the ordering preferences seen in typological data of both spoken and signed languages for the adjectives and genitives (Coons, 2022; Dryer, 2013a). Under the assumption that biases targeting meaning can come to influence conventionalized syntax (Goldin‐Meadow et al., 2008; Meir et al., 2017), our results point to a potential explanation for this typological pattern. The main difference between our results and the patterns seen in (spoken) language typology is that the postnominal preference for descriptive meanings was not stronger than the prenominal preference for possessive meaning. Interestingly, the fact that there was no difference in the strength of these preferences supports the notion that using a gesture‐based system helps limit native language influence. This is because influence from English would presumably have biased participants *against* choosing postnominal order for descriptive meanings, since adjectives (the most common instantiation of the type of descriptive properties we used) are typically prenominal in English. If English syntax was a strong influence on participants' behavior, then we would have expected the prenominal preference for possessive meanings to emerge more strongly since prenominal genitive order is common in English for the types of meanings we used.

In general, these results are in line with previous research which has found evidence for a postnominal preference for descriptive expression in silent gesture tasks (Culbertson et al., 2020; Jaffan et al., 2020), but expands on this by providing the first experimental evidence for a prenominal preference for possessive expressions. The fact that these preferences are observable in a context where no wider systematic language structure is in place suggests that prenominal possession and postnominal description orders act as default preferences in this type of context.

By contrast, Experiment 2 found no evidence for these category‐specific biases when participants were tasked with *learning* an ordering system to express the meaning of one of these expression types. Participants trained on a more natural system, where the majority order aligned with the preferred orders in Experiment 1, did not reproduce the majority orders more faithfully, or regularize these orders more readily than if they were trained on an unnatural system. Overall, learning and regularization behavior was comparable across all four conditions. Thus, there was no tendency for participants to produce more natural orders than what would be expected by chance: the overall input proportion of natural orders across all conditions was 50%, and this remained the case in participants' output across conditions. In other words, the natural order was only used more than the unnatural order if it was the majority training order for a given participant. Instead, the main results of Experiment 2 showed that participants had a general tendency to become more consistent in their use of specific word orders compared to the systems they were trained on.

To summarize, we have evidence that category‐specific ordering preferences for these two meaning types influence linguistic behavior in a specific context. In particular, when participants must choose the order of a individual linguistic item, in the absence of any knowledge of the wider system that item belongs to. Once items are taught to participants as part of a system, we no longer found evidence for these category‐specific preferences. Instead, systems that align with or deviate from natural orders were learned equally well.

#### 3.4.1 Revealing category‐specific biases

These results differ from previous work which found that preferences for basic word order conditioned on event type (i.e., gesture order aligning with SOV for extensional vs. SVO for intensional) influence behavior both in the absence of a system *and* during learning of a system (Motamedi et al., 2021). This difference is especially interesting in light of the potential conceptual parallel between the preferences found here and those found for event type. In intensional events, the existence of the object depends on the action of the verb (e.g., “gnome dreams of banana”). Similarly, some descriptive expressions (such as scalar adjectives) depend on the object they are describing for their interpretation. In both cases, there is a typological preference for ordering the dependent element (the object in the event, or adjective in the noun phrase) after the element on which it depends (the verb or noun). Conversely, objects of extensional events and possessors are both more independent of their heads; the object of an extensional verb exists independent of it, and a possessor does not typically rely on the head noun for its interpretation. There is nothing about the concept of “cup” which changes the way we interpret the existence of “vampire” in “vampire's cup.” Given this parallel, it is striking that there is a difference between our results, which demonstrate a lack of naturalness preference in learning as opposed to one‐off choice, compared to those reported in Motamedi et al. (2021), which do show this preference in both tasks.

One possibility is that the biases which affect descriptive and possessive expressions are relatively weaker than those which govern event‐type conditioning. Assuming that a category‐specific bias must be relatively strong in order to overcome evidence of a conventionalized order, this could explain why only the latter are evident in learning. However, if we believe that the semantic pressures observed here could influence syntactic ordering patterns in typology, then this would lead us to expect more languages that condition basic word order on event type compared to languages which have nonharmonic orders for adjectives and genitives. This is clearly not the case. In fact, conditioning word order on event type seems to be comparatively rare in typology, although more detailed research is needed (Flaherty et al., 2018; Napoli et al., 2017). Instead, it could be that this pattern does not survive because the pressure to regularize and converge on a consistent basic word order is also very strong, possibly stronger than the pressure to converge on a single noun phrase order. Many languages use fixed word order to signify who does what to whom. In such languages, a conventionalized basic word order is crucial for communicative purposes. This pressure could out‐compete the category‐specific preferences to condition order on event type, making it rare typologically even if it is evident in experimental contexts.

Of course, this brings up the question of how, more precisely, to link the typological prevalence of the nonharmonic order aligned with the category‐specific biases we have found here. If the biases we have uncovered in our experiments are present only during language emergence, but not during learning—either because they are too weak, or because the mechanism which underlies them is simply not active during learning—then it is somewhat surprising that the effects of these biases have persisted for so long in the face of a competing pressure for harmony.

In light of this, it is worth considering the possibility that contexts similar to language emergence might be present, albeit to a lesser degree, in more typical linguistic contexts. In other words, we might imagine that category‐specific biases more generally, or at least with these particular semantic motivations, arise whenever some level of linguistic innovation or creativity is required. Although adults tend to be fully proficient language users, there is still a surprising level of novelty and innovation employed in everyday language tasks (Christiansen & Chater, 2022). This includes instances where we integrate new lexical items into pre‐existing categories and, more specifically, during language acquisition, children often have to produce structures for which they have no direct evidence (Chomsky, 1972; Perfors, Tenenbaum, & Regier, 2011). Support for the possibility that tasks which involve some innovation might be more likely to reveal category‐specific biases can be seen in, for example, studies examining biases for noun phrase homomorphism and affix ordering (Culbertson & Adger, 2014; Martin et al., 2020; Martin et al., 2019; Saldana et al., 2021). In these experiments, ordering biases emerge when learners have been trained on part of a system but must extrapolate beyond their input in the critical task. For example, participants in Martin et al. (2020) learn that a single modifier comes after the noun, but must extrapolate beyond that to generate the relative order of multiple modifiers at test. It is possible that the biases observed for descriptive and possessive meanings in Experiment 1 would also influence linguistic behavior under such conditions. Lack of direct evidence for the full language system might cause participants to “fall back” on these category‐specific biases to a certain degree. Tasks that combine learning part of a system with a testing phase that forces participants to generalize/extrapolate beyond the system they learned in this way can also be seen as a more difficult learning task than the one we presented participants with in our experiment. The added difficulty in these types of tasks may also be key to revealing the influence of category‐specific biases in tasks that involve some system learning. We hope to examine this possibility in future studies. If participants show a tendency toward natural orders in both extrapolation and improvisation/emergence contexts, then such contexts can act as additional opportunities for category‐specific biases to influence typology over time. This would eliminate the need to claim that structures favored by category‐specific biases must be easier to learn in order for them to be observable as typological tendencies. Instead, these instances of partial innovation, where we extrapolate or generalize beyond previously learned structures, could then act to preserve the influence of category‐specific biases on language structure. This would be in line with theories stipulating that innovation and creativity have continuous effects on language, as they are common mechanisms in everyday language use (Christiansen & Chater, 2022). It is also worth noting that even if the category‐specific biases we have uncovered here are not very strong, research has shown that biases which are weak at the level of the individual can, over time, have cumulative effects on language structure, which gives rise to skewed distributions in typology (Kirby, Cornish, & Smith, 2008; Reali & Griffiths, 2009).

Before concluding, it is also worth considering a final, more deflationary possibility for why we do not see the effects of the category‐specific biases in Experiment 2. It could be that the correct meanings may not have been sufficiently activated by the stimuli used. Recall that we collected translation data from participants at the end of the trial (see summary of data presented in the Supplementary Appendix, as well as accompanying discussion). These data reveal that participants regularly provided adjective translations for the descriptive meanings, but fewer provided clear genitive (or genitive‐like) translations for the possessive meanings. As previously mentioned, in silent gesture tasks, there is not necessarily a clear mapping from gesture sequences to syntactic categories or structures. Here, accordingly, we have focused on the meanings being conveyed, rather than on the categories adjective and genitive. Therefore, evidence that participants conveyed the right meanings is more important for our purposes than evidence that particular syntactic categories were used. For example, many nongenitive translations provided still conveyed possessive meanings (e.g., “the vampire has a hat”). Moreover, the act of conveying a meaning is distinct from providing a translation of a gesture sequence into one's native language. The latter involves mental processes which may obscure participants' initial interpretations of the meanings in the study. Finally, our translation trials were not prompted by the images used to signify the possessive and descriptive meanings in the main task, rather they were prompted by the gesture videos used to express those meanings. Despite these caveats, it may be that different stimuli, or a different setup, might have led to stronger or more consistent interpretations of the meaning we intended. It may also be that more event‐like interpretations of gesture sequences (e.g., “the vampire has a hat”) were influenced by the subject‐first bias in addition to a prenominal preference for possessive expressions. We return to the way that animacy relates to both the subject‐first bias and the typological tendency for prenominal genitives in the next section.

#### 3.4.2 Why these category‐specific biases?

The experiments presented here do not directly test the underlying cause for the category‐specific preferences we have identified. Nevertheless, above, we mentioned a possible parallel between basic word order and noun phrase word order. Specifically, the idea that descriptive expressions like adjectives and the objects in intensional events depend on the head for their interpretation. The possibility that adjectives might tend to be postnominal for this reason is explicitly discussed by Culbertson et al. (2012), and is supported by the results of several experiments (Culbertson et al., 2020; Jaffan et al., 2020; Rubio‐Fernandez, Wienholz, Ballard, Kirby, & Lieberman, 2022; Weisleder & Fernald, 2009). Specifically, many common adjectives depend on the context of the noun in order for their meaning to be correctly interpreted. For example, comparing the meaning of the adjective “good” in the phrases “good pianist” and “good food” shows that the adjective denotes two very different properties of the head noun in each case. In the first phrase, it concerns how well the musician plays their instrument, whereas in the second phrase, it refers to some property of the food being considered tasty. For languages with postnominal adjectives, like Thai and Navajo, the noun has already been encountered when the adjective must be interpreted, allowing for incremental semantic processing. By contrast, users of languages that have prenominal adjectives cannot interpret the meaning of these adjective as soon as they are encountered, but need to keep it them in memory and interpret them once the head noun has given the relative context.

Evidence from typology and language processing also provide some potential explanations for the prenominal preference for possessive expressions like genitives. In particular, this preference might be rooted in the association between ownership and animacy. Prototypical possessors tend to be high on the animacy scale (Rosenbach, 2008; Silverstein, 1986; Yamamoto, 1999) and animate entities have been argued to hold a privileged position in language processing by virtue of being highly accessible (Dahl, 2008). This may lead to such referents being linearized earlier in a linguistic construction (Hawkins, 1981; McDonald, Bock, & Kelly, 1993; Tanaka, Branigan, McLean, & Pickering, 2011). The privileged position of animate entities has been suggested as an explanation for why there is a cross‐linguistic prevalence of subject initial languages in both spoken and signed languages (Dryer, 2013c; Napoli & Sutton‐Spence, 2014). This preference has also been found in silent gesture studies (Goldin‐Meadow et al., 2008; Schouwstra & de Swart, 2014), and in some young sign languages (Meir et al., 2017; Sandler, Meir, Padden, & Aronoff, 2005). With respect to genitives, several languages with variable genitive order, like English, Dutch, and Low Saxon, condition their use of prenominal versus postnominal genitive order on the animacy of the possessor, such that prenominal order is used for animate possessors and postnominal order is more likely to be used for inanimate possessors (van Bergen, 2011; Rosenbach, 2005; Strunk, 2004).^17^

We cannot directly provide evidence for any of these explanations here. However, more targeted studies examining the proposed cause of such category‐specific biases would be a fruitful way to expand our understanding of the cognitive grounding of these preferences. For example, experiments that vary the animacy status of the possessor could examine if animacy is the driving feature behind the ordering preference we find for these meanings in Experiment 1. In such experiments, we might expect high‐animacy possessors to show the strongest prenominal preference (as the category‐specific bias predicts), and lower animacy/inanimate possessors to show a less strong prenominal preference. The examples outlined above indicate that animacy has potential widespread effects in various aspects of grammar. For example, it may motivate the subject‐fist bias in basic word order, the prenominal genitive bias in nominal order, and it may also be responsible for patterns of differential object marking based on animacy (Aissen, 2003; Dahl, 2008). Despite these widespread effects, in each case, animacy is underlying the category‐specific biases—for individual sentences to have their subject first, for individual noun phrases to have their adjectives last, for individual sentences to mark (unexpected) animate objects. It is not a system‐wide bias in our terms, rather, it is a mechanism that has different ramifications for different items that span distinct parts of a linguistic system.

## 4 Conclusion

This study explored the role of category‐specific and system‐wide biases on language structure. Category‐specific biases target individual meanings, words, phrases, or utterances; system‐wide biases describe features that hold across these. Here, we targeted a case where these two types of biases appear to be in conflict: a system‐wide preference for harmony in the noun phrase (i.e., consistent order of nouns and modifiers) and category‐specific biases that lead to prenominal placement of genitives but postnominal placement of adjectives. We were interested in the contexts under which the effects of such category‐specific biases might influence language, and thus push against system‐wide preferences like harmony. In this case, we hypothesized that the category‐specific biases are semantic in nature—they influence preferences for conveying meanings (here descriptive and possessive expressions) that in turn may have consequences for order of syntactic categories (here adjectives and genitives). In Experiment 1, we found evidence for category‐specific biases favoring postnominal placement of descriptive gestures, and prenominal placement of possessor gestures when participants were asked to judge gesture order in the absence of a wider linguistic system. These results align with the evidence from typological data, where genitives, which express possession, tend to be prenominal, and adjectives, which express descriptive meanings, tend to be postnominal. However, in Experiment 2, we found that these biases did not modulate *learning* of a gesture system. Together, these results suggest that category‐specific biases may play an important role in shaping language in contexts that require innovation of expressions of meanings, rather than acquisition of conventionalized expressions of meanings. System‐wide biases in favor of harmony (and regularity more generally) may instead be active guiding forces during learning tasks. These results leave open whether there are additional contexts in which both pressures are at play. For example, tasks which involve substantial extrapolation beyond the learned input may be the locus of direct competition between the category‐specific and system‐wide biases.

## Supporting information

Supporting Information[Supplementary-material cogs70139-supl-0001]

## Notes

1 Of course, there is disagreement in the literature about whether or not the notion of subject as a category is relevant in all languages (Evans & Levinson, 2009). Below, we discuss whether this bias in fact targets the semantic category agent.

2 As mentioned briefly above, note that our definition of category‐specific bias is agnostic both to the level of abstraction of the category, and to what type of category it is. Biases of this type may concern a lower‐level, semantically defined category, like intensional versus extensional verbs, or a higher level syntactic category like adjective or object. Which level of abstract, and which type of category must be specified by the theorist. This is in principle true of system‐wide biases as well. The difference between the two types we have defined is in whether the bias targets the category itself, or the structured way in which that category is used across the language system.

3 There may even be cases where a particular cognitive mechanism leads to expressions of both types of biases, such that the same mechanism has both system‐wide and category‐specific effects depending on the type of linguistic unit it targets.

4 While these dependents display surface‐level exceptions to harmony, the typological data conflate multiple factors together, including genetic relationships among languages, historical accidents, grammaticalization patterns, and harmonic alignment with other phrasal elements (e.g., between syntactic objects and genitives). It is, therefore, crucial to establish whether there is observable behavioral evidence to support the existence of word order preferences, in addition to examining patterns in typological data.

5 Wang et al. (2025) find that participants trained on head‐dependent order in the verb phrase (e.g., VO or OV) generalize this order to the adposition phrase (e.g., Preposition‐Noun or Noun‐Postposition, respectively). However, it may be that there is enough of a system in place here to trigger the relevant bias.

6 This experiment was granted ethical approval by the School of PPLS Research Ethics Committee at the University of Edinburgh. The study was also preregistered https://osf.io/gt3fyand all associated materials and code can be viewed in the file tab at https://osf.io/xuzjr/.

7 Note that the predicted orders for descriptive meanings (postnominal) and possessive meanings (prenominal) align differently with order in English, the native language of our participants. We return to this point in the Discussion section.

8 Translation trials were mainly used to ensure that participants were paying attention to the task, and to examine if the order of elements in the English translations also reflected the proposed preferred orders for adjectives and genitives. Overviews of the translation data can be found in the Supplementary Appendix.

9 The most commonly reported L2s for participants in both conditions were French, Spanish, and German. All of these languages are reported as mainly having postnominal order for genitive meanings in WALS (Dryer, 2013b). French and Spanish are reported as mainly having postnominal adjectives (although many of the most common adjectives in these languages also appear prenominally) and German is reported as mostly having prenominal adjectives (Dryer, 2013a). Full list of L2s reported by participants in Experiment 1 can be found in the OSF repository.

10 A post‐hoc analysis examining whether participants in the possessive condition who indicated having knowledge of a language with postnominal adjective order were also more likely to choose a postnominal gesture order in the forced‐choice trial (*predicted order* ∼ *postnominal language*) revealed no such preference β = 0.22, *SE* = 0.34, *z* = 0.77, *p* = .51.

11 Note that the model for the descriptive condition included a random effect for items (i.e., the specific target image participants were exposed to). The model with a random effect for item did not converge in the possessive condition; therefore, a logistic regression model with no random effect is reported here for possessives.

12 Neither model converged with random intercepts for item.

13 This experiment was granted ethical approval by the School of PPLS Research Ethics Committee at the University of Edinburgh. The study was also preregistered at https://osf.io/d27gcand all associated materials and code can be viewed in the files tab at https://osf.io/xuzjr/.

14 The most common reported L2s were the same as for Experiment 1. Full list of L2s reported by participants in Experiment 2 can be found in the OSF repository.

15 The necessarily nonlinear nature of our entropy values made them unsuitable for analysis using linear models.

16 Conditional entropy alone could not fully capture this behavior since becoming more consistent across a whole system will also involve becoming more consistent within the context of a specific meaning, thus conflating the measure of regularization and lexically conditioned variation. Conditional entropy was defined as:

$$H\left(V|C\right)=-\sum_{c_{j}\varepsilon C}p\left(c_{j}\right)\sum_{v_{i}\varepsilon V}p\left(v_{i}|c_{j}\right)log_{2}p\left(v_{i}|c_{j}\right)$$

17 Systematic investigation of animacy as a conditioning criteria for genitive ordering is currently restricted to the spoken languages mentioned above; it is, therefore, not possible to evaluate how general this conditioning factor is outside of these, fairly closely related, spoken languages.
