# Supplementary material for: With or Without a System: How Category‐Specific and System‐Wide Cognitive Biases Shape Word Order
Source: Cogn Sci. 2025 Nov 26;49(11):e70139. doi: 10.1111/cogs.70139 (PMC12658409; doi:10.1111/cogs.70139)
Supplement: Supplementary file 1 — Supporting Information [file COGS-49-e70139-s001.pdf]

Appendix

Translation data

Participants in both experiments produced English translations, prompted by gesture videos. These trials were mainly used to ensure that participants were paying attention to the task (see exclusions based on incoherent responses), and to examine whether the order of elements in the English translations of gesture videos also reflected the proposed preferred orders for descriptive and possessive expressions.

Experiment 1 had one translation trial, whereas Experiment 2 had two. The responses were hand-coded for order of elements, either *prenominal*, *postnominal*, or *NA*. Table 1 shows the number of responses that fall each category for both descriptive and possessive meanings based on data from Experiment 1. The same data for Experiment 2 can be seen in Table 2. In an exploratory analysis of the data from Experiment 1, we examined whether the orders provided in the English translations of the gesture videos matched the ordering preferences based on video choice (i.e. did participants in the possessive condition produce more prenominal orders than is expected by chance, and did participants in the descriptive condition produce more postnominal orders than is expected by chance). The results of these analyses show that this is indeed the case for the possessive condition ($\beta = 1.17$, $SE = 0.28$, $z = 4.15$, $p < 0.001$) but not the descriptive condition ($\beta = 0.27$, $SE = 0.24$, $z = 1.13$, $p = 0.26$). Overall, more responses could be classified as either prenominal or postnominal when participants were translating descriptive meanings than when they were translating possessive meanings.

Table 1: Order of English translations given for gesture videos in Experiment 1 across both descriptive and possessive meanings.

Order	Descriptive meanings	Possessive meanings
Postnominal	71	30
Prenominal	54	87
NA	35	43
Total	160	160

The type of phrase used in the translation trials was also coded. The coding scheme for this

Table 2: Order of English translations given for gesture videos in Experiment 2 across both descriptive and possessive meanings.

Order	Descriptive meanings	Possessive meanings
Postnominal	48	15
Prenominal	111	77
NA	35	114
Total	194	206

differed between translations of descriptive and possessive videos. For possessive meanings the categories were *verb phrase* (these include possessive verb phrases like ‘the cyclops has a book’ and action-based phrases like ‘the vampire wears a hat’), *genitive* (including both the ‘s’ possessive and ‘of’ possessive), *prepositional phrase* (including ‘cyclops with a cup’ and ‘a hat on a vampire’) and *other* (including all phrases which could not be categories as any of the previous categories, such as the use of plain juxtaposition ‘hat vampire’). For descriptive meanings the categories were *adjective* (including phrases like ‘spotted hat’ and ‘stripy book’), *prepositional phrase* (like ‘hat with spots’ or ‘stripes on a book’), *verb phrase* (including ‘the cup has spots’), and *other* (including all phrases which could not be categories as any of the previous categories, such as the use of plain juxtaposition ‘scarf stripes’). Summary data of the number of translations that fell into each category for descriptive and possessive meanings in Experiment 1 can be seen in Table 3, and the same data is available for Experiment 2 in Table 4. The translations that participants gave varied quite widely in both experiments, but more videos showing descriptive meanings were straightforwardly translated using English adjective phrases than videos showing possessive meanings were translated into plain English genitives.

We found comparatively low number of pure genitive phrases in the translation data, although there were many instances of possessive verb phrases (included in translations classified as *verb phrase* responses in Tables 3 and 4). There was a difference in how the translations trials were prompted, compared to when meanings were presented to participants during the main part of the experiment in that the translation trials were prompted by the gesture *videos* rather than the image grid used to elicit in the main task. It is possible that the lack of contrast between different possessive contexts that those distractors provide contributed to the low number of straightforwardly genitive

translations that we got from participants. Our guess is that seeing these dynamic gestures in isolation may well have prompted more verb-phrase responses in general as they involve movements to signify objects which can be interpreted as actions, rather than nouns (e.g. the act of putting on a hat is used to signify the meaning 'hat'). This issue is not quite as strong for descriptive meanings since no actor who could perform the act in the gesture is included in the meanings, even if the same gestures are used to signify objects. It is therefore hard to say whether these translation can be interpreted as a measure for the internal representations of meaning that participants activated during the main task. Perhaps for these reasons, this type of translation task data is not typically reported in silent gesture experiments. If anything, participants are asked to produce descriptions of stimulus images, which are then coded in terms of the order of information provided, and not in terms of the syntax used (e.g., 'Agent-Patient-Action' not 'SOV' in Goldin-Meadow, So, Özyürek, & Mylander, 2008). That said, if we subset the data to only those participants who gave clear genitive translation in Experiment 1, then we find that all of those participants selected the prenominal gesture order, suggesting that the prenominal preference remains in these few instances.

Table 3: Classification of English translations given for gesture videos in Experiment 1 across both descriptive and possessive meanings.

Class	Descriptive meanings	Possessive meanings
Adjective	54	NA
Genitive	NA	10
Preposition	44	21
Verb phrase	1	54
Other	61	75
Total	160	160

References

Goldin-Meadow, S., So, W. C., Özyürek, A., & Mylander, C. (2008). The natural order of events: How speakers of different languages represent events nonverbally. *Proceedings of the National Academy of Sciences*, *105*(27), 9163–9168.

Table 4: Classification of English translations given for gesture videos in Experiment 2 across both descriptive and possessive meanings.

Class	Descriptive meanings	Possessive meanings
Adjective	106	NA
Genitive	NA	4
Preposition	37	41
Verb phrase	3	16
Other	48	145
Total	194	206
